# Hepatoprotective effect of *Paederia foetida* on paracetamol-induced hepatobiliary disease in rats via alteration of inflammatory and Nrf2/HO-1 pathway

**DOI:** 10.1590/acb409925

**Published:** 2025-08-08

**Authors:** Zhenzhen Feng, Jianyin Li

**Affiliations:** 1 Dazhou Vocational College of Chinese Medicine - College of Medical Technology - Dazhou - China.; 2 Jinan Central Hospital affiliated to Shandong First Medical University - Department of Hepatobiliary Surgery - Jinan - China.

**Keywords:** Hepatobiliary Disease, Inflammation, Antioxidants

## Abstract

**Purpose:**

To scrutinize the hepatoprotective effect of *Paederia foetida* against paracetamol-induced hepatobiliary in rats.

**Methods:**

Rats were received the oral administration of paracetamol (3 mg/kg) empty stomach for the induction of hepatobiliary in rats except normal rats. the rats were received the oral administration of *P. foetida*. The hepatic, non-hepatic, antioxidant, and inflammatory parameters were estimated, as well as the mRNA expression.

**Results:**

*Paederia foetida* treatment remarkably suppressed the level of hepatic parameters such as alkaline phosphatase, alanine transaminase, aspartate aminotransferase; and non-hepatic parameters like creatinine, total protein, bilirubin, and blood urea nitrogen level at dose dependent manner. It also altered the level of antioxidant parameters such as lipid peroxidation, superoxide dismutase, glutathione peroxidase, glutathione, and catalase in the serum, as well as hepatic tissue, and also suppressed the level of cytokines such as tumor necrosis factor-α, interleukin-1β, interleukin-6; and inflammatory parameters such as cyclooxygenase-2, prostaglandin, and nuclear factor kappa B, respectively. *Paederia foetida* also altered the level of nuclear factor erythroid 2-related factor 2 (Nrf2) and heme oxygenase-1 (HO-1).

**Conclusion:**

*Paederia foetida* exhibited the hepatoprotective effect against paracetamol-induced hepatobiliary disease in rats via inflammatory and Nrf2/HO-1 pathway.

## Introduction

Liver is the vital organ of the body that plays a crucial role during the metabolism and elimination and detoxification of toxic substances^1^. The liver tissue is commonly affected via drugs and environmental pollutants, since all of the substances increase the burden of the organ and can weaken and damage it, eventually leading to diseases such as cirrhosis or hepatitis^2^,^3^. Hepatic injury and its related alteration of metabolic function are still major health burden globally. Unfortunately, available treatment for the hepatic damage are synthetic drugs, but synthetic drugs have limitation and serious side effects. With the constraints and adverse effects associated with synthetic drugs, there is a burgeoning interest in investigating traditional herbal medicines that possibly have hepatoprotective effects^4^-^6^.

The common side effect of paracetamol (acetaminophen), analgesic and antipyretic drugs is the liver toxicity. Paracetamol undergoes hepatic metabolism to form sulfate and glucuronide conjugates, which are then excreted from the body^2^. Paracetamol induces hepatotoxicity primarily through the formation of its reactive metabolite, N-acetyl-p-benzoquinone imine (NAPQI), which leads to glutathione depletion and oxidative stress within the tissue^7^. Paracetamol induces hepatic toxicity by generating toxic metabolites during its metabolism via cytochrome P450 enzymes. NAPQI, one of these metabolites, forms covalent bonds with cysteine groups on proteins, resulting in the formation of 2-(cysteine-S-yl) acetaminophen adducts^7^,^8^. Paracetamol-induced hepatotoxicity requires the introduction of cytochrome or the depletion of hepatic glutathione^7^,^9^. Despite enormous advancements in modern medicine, various treatment available for the cure of liver dysfunction is inadequate, and various herbal extracts contain formulation used for the regeneration of hepatic cells and also for the treatment of liver against the damage^7^,^9^.

Hepatobiliary diseases encompass a wide range of conditions affecting the liver, bile ducts, and gallbladder, including hepatitis, cirrhosis, cholestasis, and hepatocellular carcinoma. These disorders are characterized by chronic inflammation, which plays a central role in liver injury, fibrosis, and progression to end-stage liver disease^10^,^11^. The complex interplay between various cellular and molecular mechanisms contributes to the pathogenesis of these conditions, making them challenging to treat and manage effectively.

The inflammatory process in hepatobiliary diseases involves key mediators such as cytokines (tumour necrosis factor-α-TNF-α, interleukin-IL-6, and IL-1β), chemokines, and reactive oxygen species (ROS), which initiate and perpetuate liver damage. These pro-inflammatory factors are produced by various cell types, including hepatocytes, Kupffer cells, and infiltrating immune cells^11^-^13^. The sustained release of these mediators leads to a chronic state of inflammation, which can persist for years or even decades, gradually eroding liver function and structure. This chronic inflammation leads to hepatocyte injury, activation of Kupffer cells (liver macrophages), and stimulation of hepatic stellate cells (HSCs), which are major drivers of liver fibrosis. Hepatocyte injury triggers a cascade of events, including the release of damage-associated molecular patterns that further amplify the inflammatory response. Activated Kupffer cells secrete additional pro-inflammatory cytokines and chemokines, recruiting more immune cells to the site of injury. HSCs, when activated, transform into myofibroblast-like cells that produce excessive amounts of extracellular matrix proteins, leading to the accumulation of fibrous tissue and the development of liver fibrosis^14^,^15^.

The nuclear factor erythroid 2-related factor 2 (Nrf2) pathway emerges as a critical regulator of the cellular antioxidant response in hepatobiliary diseases. This transcription factor plays a pivotal role in maintaining cellular redox homeostasis and protecting against oxidative stress-induced damage. Under normal conditions, Nrf2 is sequestered in the cytoplasm by its inhibitor, Kelch-like ECH-associated protein 1 (Keap1), which targets Nrf2 for ubiquitination and subsequent proteasomal degradation. Under oxidative stress conditions, Nrf2 dissociates from its inhibitor Keap1, translocates to the nucleus, and activates the expression of various antioxidant and cytoprotective genes, including heme oxygenase-1 (HO-1). This process occurs through the binding of Nrf2 to antioxidant response elements (AREs) in the promoter regions of its target genes. The activation of the Nrf2 pathway represents a crucial adaptive mechanism that allows cells to cope with oxidative and electrophilic stress, which are common features of hepatobiliary diseases^15^,^16^.

The Nrf2/HO-1 pathway plays a dual role in the liver by both reducing oxidative stress and modulating inflammatory responses. Nrf2 activation downregulates pro-inflammatory cytokine production and inhibits the activation of NF-κB, a key transcription factor involved in the inflammatory response. This anti-inflammatory effect is mediated, in part, by the induction of HO-1, which catalyzes the degradation of heme to biliverdin, carbon monoxide, and free iron. These byproducts, particularly carbon monoxide and biliverdin (which is rapidly converted to bilirubin), possess potent anti-inflammatory and antioxidant properties. Additionally, by upregulating HO-1 and other antioxidant genes, Nrf2 reduces ROS levels, limiting oxidative damage and lipid peroxidation in liver cells. The antioxidant enzymes induced by Nrf2, such as glutathione S-transferases, NAD(P)H:quinone oxidoreductase 1, and glutamate-cysteine ligase, work in concert to neutralize ROS and other toxic metabolites. This coordinated antioxidant response helps maintain the redox balance within hepatocytes and other liver cells, preventing oxidative stress-induced cellular damage and death^14^-^16^.

*Paederia foetida* Linn, popularly known as Chinese fever vine, has multiple pharmacological potential against various diseases^17^. A rich source of glycosides, flavonoids and terpenoids, *P. foetida* L. has smell due to presence of methyl mercaptane. It has antidiabetic, antitoxic, antitumor, anti-inflammatory, and anti-arthritic effects^17^-^19^, and helps in the maintenance of mild hypercholesterolaemia. *Paederia foetida* L. already has antioxidant and anti-inflammatory effects^18^,^20^. The present experimental study aimed to investigate the hepatoprotective effect of *P. foetida* L. against paracetamol-induced hepatobiliary disease in rats and elucidate the underlying mechanism, focusing on its antioxidant and anti-inflammatory properties.

## Methods

### Plant material

The leaves of the *P. foetida* L. were collected from the departmental herbal garden and authenticated by the Prof. Yie Li, botanist with identification number (ACHB-73849), and a specimen sample was submitted to the institution for further references.

### Preparation of the extract

The leaves of *P. foetida* Linn were dried in shade for seven days and then pulverized into coarse powder. The powdered leaves were extracted with methanol using a Soxhlet apparatus for 24 hours at 80°C. After the extraction process was completed, the solvent was completely removed from the extract sample. The removal of water from the extract was achieved through freeze drying, resulting in an extract sample with a yield of 24.5% (w/w). The extract was subsequently stored in a refrigerator, and the desired quantity was dissolved in 10 mL of distilled water before being used in the current experimental study.

### Experimental animal

For the current experimental study, Wistar albino rats of both sexes weighing between 200-220 g were utilized. The rats were obtained from the animal house facility and housed under standard experimental conditions, including temperature of 21 ± 5°C, relative humidity of 70 ± 5%, and a 12-hour light-dark cycle. They were provided with standard chow (Chun Li Feed co.) and water *ad libitum* throughout the study. The current research was carried out in the College of Pharmacy, Dazhou Vocational College of Chinese Medicine, Dazhou 635000, China in the month of December 2023.

### Paracetamol-induced hepatic tissue damage

The animals were randomly divided into six groups, each consisting of six rats, as outlined below. For the paracetamol-induced liver damage model, the rats were divided into six groups, with each group containing six rats. The experimental groups were as it follows:

- Group I: normal control rats (rats received the oral administration of 1% of aqueous solution of carboxymethyl cellulose-CMC for 14 days);
- Group II: paracetamol-induced liver damage (rats received 3 mg/kg of paracetamol);
- Group III: paracetamol-induced liver damage received *P. foetida* L. (25 mg/kg);
- Group IV: paracetamol-induced liver damage received *P. foetida* L. (50 mg/kg);
- Group V: paracetamol-induced liver damage received *P. foetida* L. (100 mg/kg);
- Group VI: paracetamol-induced liver damage received silymarin (25 mg/kg).

The dose selection was used on the previous reported method^18^,^21^,^22^. At the end of the experimental protocol, the rats were anesthetized using ether anesthesia. Blood samples were collected by puncturing the retro-orbital plexus and collecting the blood in ethylenediaminetetraacetic acid (EDTA) tubes. Following blood collection, all rats in each group were euthanized via cervical dislocation. Hepatic tissue was then removed and divided into two portions. One portion was stored at -80°C for the estimation of biochemical parameters, while the other portion was used for reverse-transcription polymerase chain reaction (RT-PCR) investigation.

### Hepatic, non-hepatic and renal parameters

Hepatic parameters such as alanine transaminase (ALT), aspartate aminotransferase (AST), alkaline phosphatase (ALP), low-density lipoprotein (LDL), gamma-glutamyl transferase (GGT), total bilirubin, and albumin; and renal parameters viz., urea, uric acid, and creatinine were estimated using the enzyme-linked immune sorbent assay (ELISA) kits following the manufacture instruction (Biosystem S.A., Spain).

### Glucose

Glucose kit (Roche Diagnostic, United States of America) was used for the estimation of glucose parameters.

### Lipid parameters

Lipid parameters such as triglyceride (TG), total cholesterol (TC), LDL, and high-density lipoprotein (HDL) were estimated by the available reagent kits following the manufacture instruction (Accurex).

### Oxidative stress parameters

Oxidative stress parameters such as lipid peroxidation (LPO), glutathione peroxidase (GPx), glutathione (GSH), superoxide dismutase (SOD), and glutathione S-transferase (GST) were estimated using the ELISA kits according to the manufacturer’s instructions (Biosystem S.A., Spain).

### Pro-inflammatory cytokines and inflammatory parameters

Pro-inflammatory cytokines such as interleukin-1β (IL-1β), 2, 6, 7, 10, 17, 18 and TNF-α; and inflammatory parameters cyclooxygenase-2 (COX-2), prostaglandin (PGE_2_), transforming growth factor beta (TGF-β) and nuclear factor kappa B (NF-κB) were estimated via the ELISA kits following the manufacture instructions (R&DSystems, United States of America).

### Reverse transcriptase-polymerase chain reaction

Total RNA from tissues was isolated using the TRIzol reagent according to the manufacturer’s instructions. The quantity and purity of the RNA were assessed spectrophotometrically (*e.g.*, NanoDrop), measuring the absorbance at 260 and 280 nm. Reverse transcription was performed using the high-capacity cDNA Reverse transcription kit with 1 µg of total RNA as input. The reaction mix (20 µL end value reaction) usually included: 1 µg of RNA, 1× reverse transcription buffer, dNTP mix (1 mM each), Random primers or oligo(dT), reverse transcriptase enzyme, RNase inhibitor and the following reverse transcription thermal cycling conditions were applied: 25°C for 10 min, 37-42°C for 60 min and 85°C for 5 min to stop the reaction, respectively. The cDNA was then used as template for PCR amplification with the gene specific primers. A typical PCR set up (25-50 µL volume) contained: 1-2 µL of cDNA template, 1×PCR buffer, dNTPs (200 µM each), forward and reverse primers (0.2-0.5 µM each), Taq DNA polymerase (0.5-1.0 units), MgCl2 (1.5-2.5 mM).

For most experiments the following thermal cycling conditions were employed (optimized according to the primer set):

- Initial denaturation: 94-95°C for 3-5 min, 30-40 cycles;
- Denaturation: 94-95°C for 30 sec;
- Annealing: 55-65°C for 30 sec.

PCR products were fractionated on a 1.5-2% agarose gel, stained with ethidium bromide or safe alternative (e.g., SYBR Safe) and were visualized using a ultraviolet light source. Band sizes were referred to a DNA ladder. Expression of target genes was then normalized with a reference gene (such as β-actin) and quantified by densitometry analysis. The primers sequences for IL-1β, TNF-α, COX-2 and iNOS are listed in Table 1.

**Table 1:** List of gene primer sequences.

S. No	Gene	Sequence	
		Reverse (50-30)	Forward (50-30)
1	Caspase-3	ACACAAGCCCATTTCAGGGT	GGAGCTTGGAACGCGAAGAA
2	Bcl-2	TGACATCTCCCTGTTGACGC	ACTCTTCAGGGATGGGGTGA
3	Bax	CAGTTGAAGTTGCCGTCTGC	AGGACGCATCCACCAAGAAG
4	HO-1	ATGTGCCAGGCATCTCCTTC	GTAAATGCAGTGTTGGCCCC
5	Nrf2	TGTCCTGCTGTATGCTGCTT	TTGTAGATGACCATGAGTCGC
6	NF-κB p65	GGTCCCGTGTAGCCATTGAT	CCTCATCTTTCCCTCAGAGCC
7	NQO-1	TCCTTGTGGAACAAAGGCGA	GGCCATCATTTGGGCAAGTC
8	β-actin	CGCAGCTCAGTAACAGTCCG	AGGAGTACGATGAGTCCGGC

HO-1: heme oxygenase-1; Nrf2: nuclear factor erythroid 2-related factor 2; NF-κB: nuclear factor kappa B.

### Statistical analysis

The results of the current study were analyzed using a one-way analysis of variance (ANOVA) test with Dunnett’s t-test for *post hoc* analysis. GraphPad Prism 8.0 software (St. Louis, United States of America) was utilized for statistical analysis. All data were presented as mean ± standard error of the mean, and *p* < 0.05 was considered statistically significant.

## Results

### Hepatic and non-hepatic parameters and glucose level

**Figure 1:**
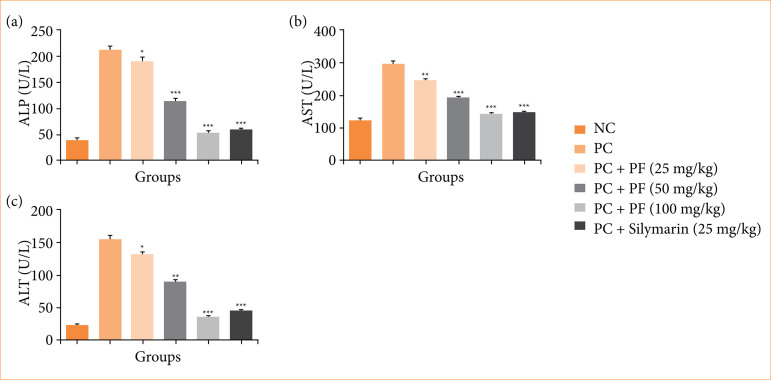
Effect of *Paederia foetida* L. on the hepatic parameters against the paracetamol-induced hepatobiliary in rats. **(a)** alkaline phosphatase (ALP), **(b)** aspartate aminotransferase (AST), and **(c)** alanine transaminase (ALT). Data are presented as mean ± standard error of the mean (n = 6). *p < 0.05, ** p < 0.01, and *** p < 0.001 were considered as significant, more significant and extreme significant. *p < 0.05: significant; **p < 0.01: more significant; ***p < 0.001: extreme significant; NC: normal control; PC: paracetamol; PF: *Paederia foetida* L.

Source: Elaborated by the authors.

Paracetamol-induced hepatobiliary rats revealed the boosted level of hepatic parameters like ALP (Fig. 1a), AST (Fig. 1b), and ALT (Fig. 1c), and *PF* treatment significantly (*p* < 0.001) repressed the level of hepatic parameters.

**Figure 2:**
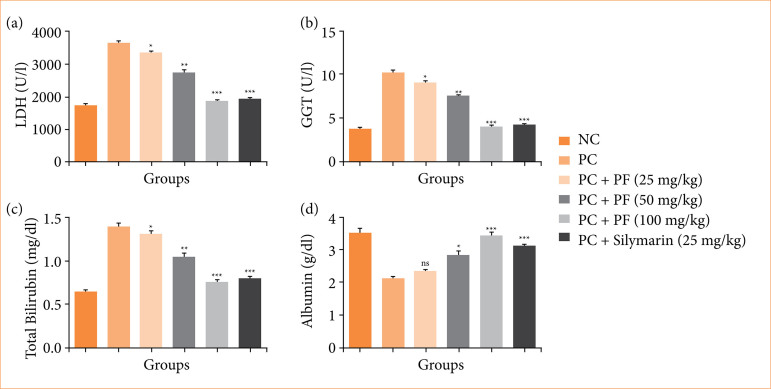
Effect of *Paederia foetida* L. on the non-hepatic parameters against the paracetamol-induced hepatobiliary in rats. **(a)** low-density lipoprotein, **(b)** gamma-glutamyl transferase (GGT), **(c)** total bilirubin, and **(d)** albumin. Data are presented as mean ± standard deviation (n = 6). *p < 0.05: significant; **p < 0.01: more significant; ***p < 0.001: extreme significant; NC: normal control; PC: paracetamol; PF: *Paederia foetida* L.

Source: Elaborated by the authors.

The level of LDH (Fig. 2a), GGT (Fig. 2b), and total bilirubin (Fig. 2c) boosted, and the level of albumin (Fig. 2d) suppressed in the paracetamol-induced hepatobiliary group rats. PF treatment remarkably restored the level of non-hepatic parameters.

**Figure 3:**
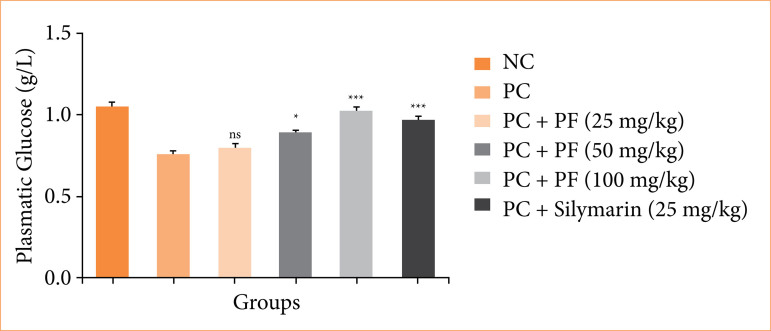
Effect of *Paederia foetida* L. on the plasmatic glucose against the paracetamol-induced hepatobiliary in rats. Data are presented as mean ± standard deviation (n = 6). *p < 0.05: significant; **p < 0.01: more significant; ***p < 0.001: extreme significant; NC: normal control; PC: paracetamol; PF: *Paederia foetida* L.

Source: Elaborated by the authors.

During the hepatic injury, the level of glucose reduced due to expansion of disease. Paracetamol-induced hepatobiliary group rats exhibited the suppressed level of plasmatic glucose (Fig. 3), and *PF* treatment significantly improved the level of plasmatic glucose.

### Renal parameters

**Figure 4:**
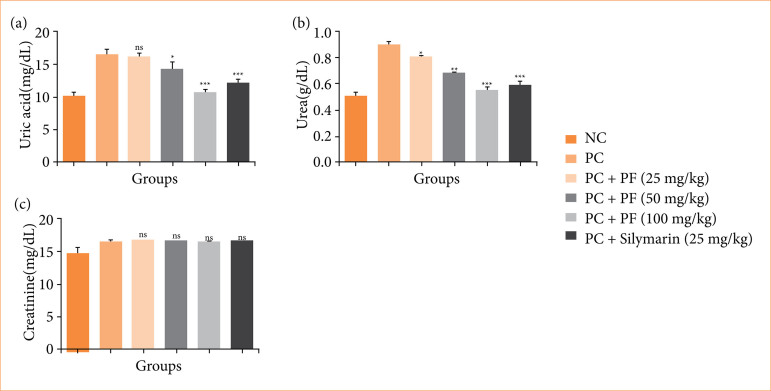
Effect of *Paederia foetida* L. on the renal parameters against the paracetamol-induced hepatobiliary in rats. **(a)** urea, **(b)** uric acid, and **(c)** creatinine. Data are presented as mean ± standard deviation (n = 6). *p < 0.05: significant; **p < 0.01: more significant; ***p < 0.001: extreme significant; NC: normal control; PC: paracetamol; PF: *Paederia foetida* L.

Source: Elaborated by the authors.

Paracetamol-induced hepatobiliary group rats showed the modulated level of uric acid (Fig. 4a), urea (Fig. 4b), and creatinine (Fig. 4c), and *PF* treatment altered the level of renal parameters.

### Lipid parameters

**Figure 5:**
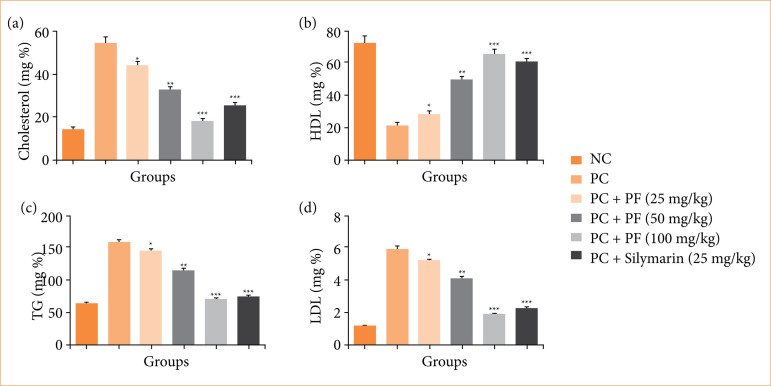
Effect of *Paederia foetida* L. on the lipid parameters against the paracetamol induced hepatobiliary in rats. **(a)** cholesterol, **(b)** high-density lipoprotein (HDL), **(c)** triglyceride (TG) and **(d)** low-density lipoprotein (LDL). Data are presented as mean ± standard deviation (n = 6). *p < 0.05: significant; **p < 0.01: more significant; ***p < 0.001: extreme significant; NC: normal control; PC: paracetamol; PF: *Paederia foetida* L.

Source: Elaborated by the authors.

Paracetamol-induced hepatobiliary group rats displayed the altered level of lipid parameters such as cholesterol (Fig. 5a), HDL (Fig. 5b), TG (Fig. 5c), and LDL (Fig. 5d), and *PF* treatment significantly (*p* < 0.001) restored the level of lipid parameters.

### Oxidative stress parameters

**Figure 6:**
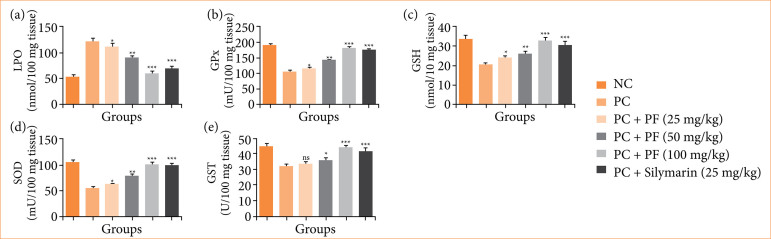
Effect of *Paederia foetida* L. on the oxidative stress parameters against the paracetamol-induced hepatobiliary in rats. **(a)** lipid peroxidation (LPO), **(b)** glutathione peroxidase (GPx), **(c)** glutathione (GSH), **(d)** superoxide dismutase (SOD), and **(e)** glutathione S-transferase (GST). Data are presented as mean ± standard deviation (n = 6). *p < 0.05: significant; **p < 0.01: more significant; ***p < 0.001: extreme significant; NC: normal control; PC: paracetamol; PF: *Paederia foetida* L.

Source: Elaborated by the authors.

The level of LPO (Fig. 6a) boosted, and the level of GPx (Fig. 6b), GSH (Fig. 6c), SOD (Fig. 6d), and GST (Fig. 6e) reduced in the paracetamol-induced hepatobiliary group rats. *PF* treatment significantly (*p* < 0.001) altered the level of oxidative stress parameters.

### Cytokines and inflammatory parameters

**Figure 7:**
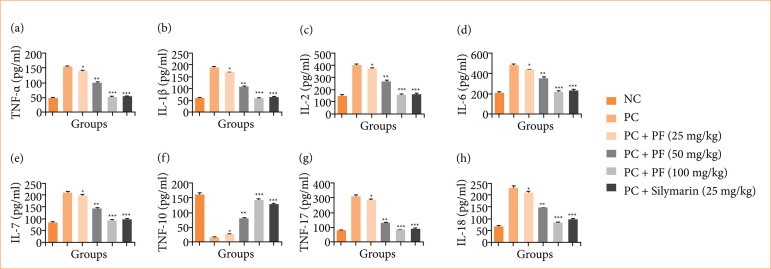
Effect of *Paederia foetida* L. on the cytokines parameters against the paracetamol-induced hepatobiliary in rats. **(a)** tumor necrosis factor- (TNF)-α, **(b)** interleukin (IL)-1β, **(c)** IL-2, **(d)** IL-6, **(e)** IL-7, **(f)** IL-10, **(g)** IL-17, and **(h)** IL-18. Data are presented as mean ± standard deviation (n = 6). *p < 0.05: significant; **p < 0.01: more significant; ***p < 0.001: extreme significant; NC: normal control; PC: paracetamol; PF: *Paederia foetida* L.

Source: Elaborated by the authors.

The level of cytokines such as TNF-α (Fig. 7a), IL-1β (Fig. 7b), IL-2 (Fig. 7c), IL-6 (Fig. 7d), IL-7 (Fig. 7e), IL-10 (Fig. 7f), IL-17 (Fig. 7g), and IL-18 (Fig. 7h) altered in the paracetamol-induced hepatobiliary group rats, and *PF* treatment significantly (*p* < 0.001) restored the level of cytokines.

**Figure 8:**
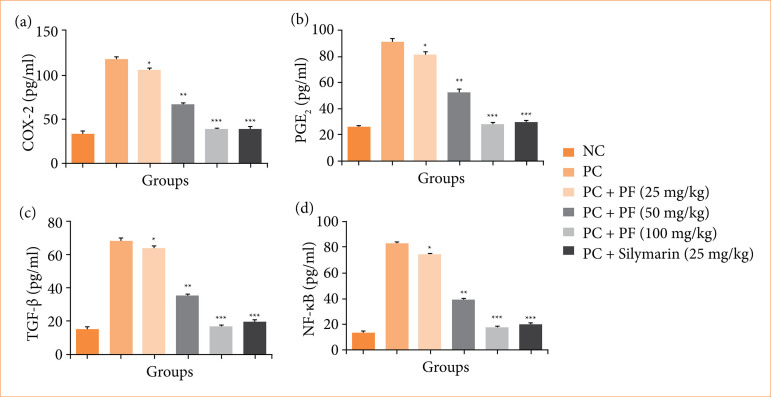
Effect of *Paederia foetida* L. on the inflammatory parameters against the paracetamol-induced hepatobiliary in rats. **(a)** cyclooxygenase-2 (COX-2), **(b)** prostaglandin (PGE2), **(c)** transforming growth factor beta (TGF-β), and **(d)** nuclear factor kappa B (NF-κB). Data are presented as mean ± standard deviation (n = 6).

Paracetamol-induced hepatobiliary group rats showed the boosted level of inflammatory parameters such as COX-2 (Fig. 8a), PGE_2_ (Fig. 8b), TGF-β (Fig. 8c), and NF-κB (Fig. 8d), and *PF* treatment significantly (*p* < 0.001) suppressed the level of inflammatory parameters.

### mRNA expression

**Figure 9:**
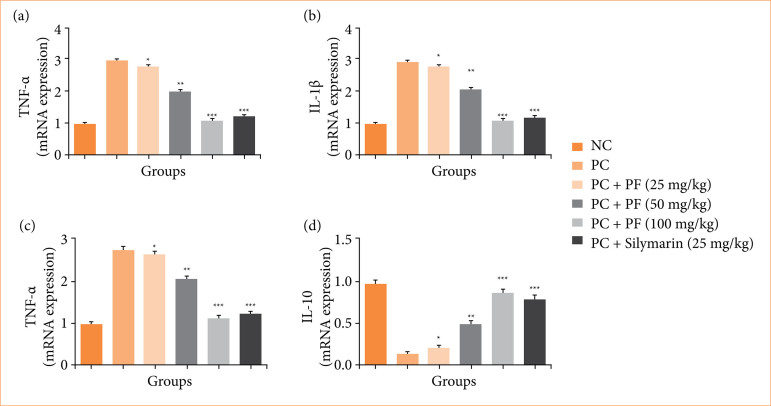
Effect of *Paederia foetida* L. on the mRNA expression of cytokines against the paracetamol-induced hepatobiliary in rats. (a) tumor necrosis factor (TNF)-α, (b) interleukin (IL)-1β, (c) IL-6, and (d) IL-10. Data are presented as mean ± standard deviation (n = 6). *p < 0.05: significant; **p < 0.01: more significant; ***p < 0.001: exto

Source: Elaborated by the authors

**Figure 10:**
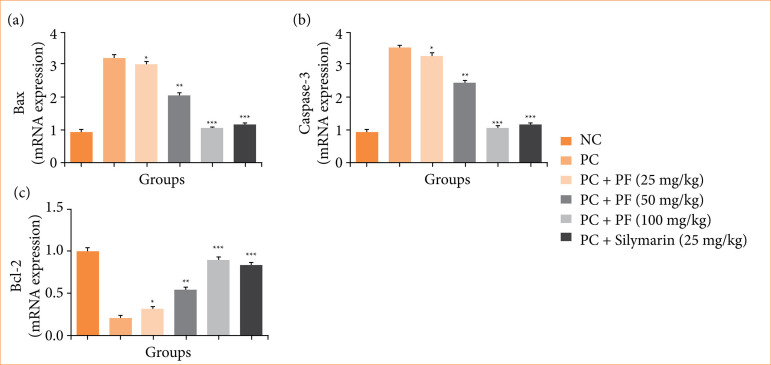
Effect of *Paederia foetida* L. on the mRNA expression of apoptosis against the paracetamol-induced hepatobiliary in rats. **(a)** Bax, **(b)** caspase, and **(c)** Bcl-2. Data are presented as mean ± standard deviation (n = 6). *p < 0.05: significant; **p < 0.01: more significant; ***p < 0.001: extreme significant; NC: normal control; PC: paracetamol; PF: *Paederia foetida* L

Source: Elaborated by the authors.

Paracetamol-induced hepatobiliary group rats exhibited the altered mRNA expression of cytokines viz., TNF-α (Fig. 9a), IL-1β (Fig. 9b), IL-6 (Fig. 9c), and IL-10 (Fig. 9d); and apoptosis such as Bax (Fig. 10a), caspase-3 (Fig. 10b), and Bcl-2 (Fig. 10c). *PF* treatment modulated the mRNA expression of cytokines and apoptosis.

**Figure 11:**
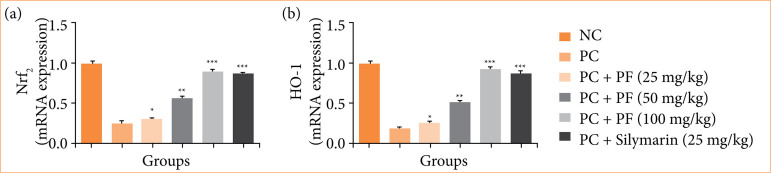
*p < 0.05: significant; **p < 0.01: more significant; ***p < 0.001: extreme significant; NC: normal control; PC: paracetamol; PF: *Paederia foetida*

Source: Elaborated by the authors.

The mRNA expression of Nrf2 (Fig. 11a), and HO-1 (Fig. 11b) reduced in the paracetamol-induced hepatobiliary group rats, and *PF* treatment significantly (p < 0.001) restored the level of Nrf_2_ and HO-1.

**Figure 12:**
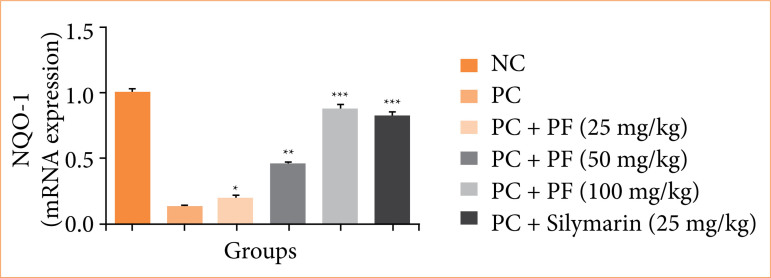
Effect of *Paederia foetida* L. on the mRNA expression of NQO-1 against the paracetamol-induced hepatobiliary in rats. Data are presented as mean ± standard deviation (n = 6). *p < 0.05: significant; **p < 0.01: more significant; ***p < 0.001: extreme significant; NC: normal control; PC: paracetamol; PF: *Paederia foetida* L.

Source: Elaborated by the authors.

**Figure 13:**
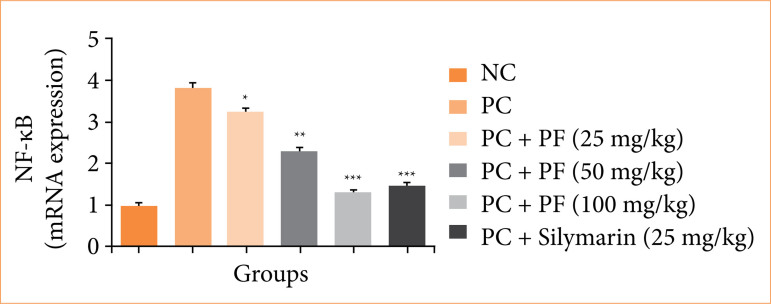
Effect of *Paederia foetida* L. on the mRNA expression of nuclear factor kappa B (NF-κB) against the paracetamol-induced hepatobiliary in rats. Data are presented as mean ± standard deviation (n = 6).*p < 0.05: significant; **p < 0.01: more significant; ***p < 0.001: extreme significant; NC: normal control; PC: paracetamol; PF: Paederia foetida L.

Source: Elaborated by the authors.

## Discussion

Paracetamol-induced liver injury serves as a model for assessing the hepatoprotective effects of drugs, as it is characterized by xenobiotic-induced hepatotoxicity^23^. It is well documented that hepatic tissue is considered as the significant tissue among the other organs in the body. Hepatic tissue provides the various physiological functions in the body such as excretory and secretory function, protein synthesis, homeostasis maintenance, and finally storage of nutrients^24^,^25^. Hepatic tissue also protects the body from the side effect of various drugs and xenobiotic.

Previous studies suggested that the chemical solvent that induces the hepatic injury like to that produced it via viral hepatitis^26^,^27^. As per our best knowledge, this is the first time hepatoprotective effect of *P. foetida* L. were performed against paracetamol-induced hepatobiliary disease in rats. In this study, silymarin was utilized as the positive control, despite potential differences in its underlying mechanism of action compared to the test compounds. Silymarin is a polyphenolic flavonoid derived from the seeds and fruits of *Silybum marianum* 28. Previous studies show that silymarin and S. marianum extract demonstrate the hepatoprotective effect via antioxidant mechanism^29^.

The hepatic tissue receives the paracetamol (foreign toxin), which turns into the two free radicals. These free radicals induce the oxidation or production of unsaturated lipids. The oxidation of unsaturated lipids starts the induction of hepatic affections^30^. Moreover, various compounds are showed the beneficial effect against the hepatic dysfunction induced by paracetamol, via applying their protective effect either via antioxidant potential or attenuation of paracetamol-derived free radical production^31^,^32^. We found that the paracetamol induces the significant alteration in the status of oxidant/antioxidant, which was categorized via considerable boost the level of LPO in the hepatic tissue and marked reduction of hepatic GPx, GSH, SOD, and GST activities. These finding are in accordance with the previous investigation and confirm the hepatoprotective effect of *P. foetida* L.

Previous investigation suggest that the antioxidants prevent the free radical generation, ROS formation and oxidative stress induced liver toxicity via scavenging the free radicals and ROS^33^,^34^. Flavonoids play an important role in the scavenge the free radicals and antioxidant in biological systems^35^. Previous investigations suggested that the flavonoids have various pharmacological effects such as antibacterial, anti-diabetic, antioxidant, anti-inflammatory, antimutagenic, and anticancer effects^36^,^37^. *Paederia foetida* L. is a rich source of the flavonoisd.

In the current experimental study, we investigated the hepatoprotective effect of *P. foetida* L. against paracetamol-induced liver injury. Paracetamol provoked the liver injury in the rats, and hepatic injury was scrutinized via assessing the activities of hepatic parameters such as ALP, ALT, and AST (hepatic maker)^38^,^39^. The paracetamol treatment increased the level of hepatic parameters in the circulation due to leakage from the hepatic tissue. The elevated levels of AST are typically accompanied by an increase in the levels of ALT. ALT plays a crucial role in the conversion of amino acids to keto acids^32^,^38^,^40^. *Paederia foetida* L. treatment considerably attenuated the boosted level of serum hepatic markers. The result suggested that *P. foetida* L. extract protect the hepatocytes and integrity of membrane from paracetamol-induced leakage into the serum marker into the circulation. These alterations can be interpreted as functional improvements in hepatocytes and may be induced by the accelerated regeneration of parenchymal cells. Studies suggested that the increase level of bilirubin and ALP are responsible for damaging the hepatic cell. The level of ALP boosted in the serum due to rise the synthesis in the presence of enhancing biliary pressure^41^. In this study, we have found that *P. foetida* L. treatment suppressed the level of bilirubin and ALP due to its antioxidant nature, which may protect the hepatic cell from the injury induced via paracetamol.

The reduction in the albumin in the serum is a hallmark of hepatic diseases^42^. Reports suggested that a decrease in serum albumin levels is associated with an increase in the levels of beta and gamma globulins due to the production of IgM and IgG. Additionally, elevated levels of bilirubin in the serum indicate impaired liver tissue function in storing bilirubin^42^,^43^. Liver parenchymal cells transport bilirubin, in which it undergoes conjugation with glucuronic acid facilitated by the enzyme glucuronyl-transferase. This conjugated bilirubin is then excreted into bile through the process of glucuronidation^44^,^45^. Hepatic parenchymal cells injury leads to boost the level of bilirubin in the serum in paracetamol-induced hepatobiliary in the rats, and *PF* treatment altered the level of bilirubin and albumin in the serum suggesting the protective effect against hepatobiliary.

NF-κB is well recognized for its pivotal role in regulating the expression of various genes such as TNF-α, IL-1β, and COX-2. These genes are implicated in inflammation, tumorigenesis, and autoimmune diseases. Specifically, TNF-α, a pro-inflammatory cytokine, is instrumental in the inflammatory response and can exacerbate hepatocyte injury and apoptosis in paracetamol-induced hepatotoxicity^30^,^46^. IL-1β is another pro-inflammatory cytokine that is implicated in the inflammatory cascade following paracetamol overdose. It can exacerbate liver injury by promoting inflammation and cell death^31^. IL-2 is primarily involved in regulating immune responses. While its specific role in paracetamol-induced hepatobiliary injury is not well-defined, it may contribute to the overall inflammatory response. IL-6 is a pleiotropic cytokine involved in inflammation and immune regulation. It is known to increase in response to paracetamol overdose and may contribute to hepatocyte injury and inflammation. IL-7 is important for T-cell development and function^47^. Its role in paracetamol-induced hepatobiliary injury is not extensively studied, but it may modulate immune responses during liver injury. IL-10 is an anti-inflammatory cytokine that can limit the inflammatory response and protect against tissue damage. Its elevation in paracetamol overdose may represent a compensatory mechanism to counteract excessive inflammation. IL-17 is associated with inflammatory responses and tissue damage in various diseases. Its role in paracetamol-induced hepatobiliary injury is not fully elucidated but may contribute to inflammation and liver injury^47^,^48^. IL-18 is a pro-inflammatory cytokine that can amplify the immune response. Its involvement in paracetamol-induced hepatotoxicity is implicated in promoting inflammation and hepatocyte injury. Paracetamol-induced hepatobiliary rats exhibited the altered level of cytokines, and *PF* treated group rats restored the level of cytokines.

Both Nrf2 and HO-1 are integral components of the cellular defense system against oxidative stress and inflammation^49^. Nrf2 and HO-1 play essential roles in protecting against paracetamol-induced hepatobiliary injury by activating antioxidant and cytoprotective pathways, reducing oxidative stress, and inflammation. Altering the activity of Nrf2 and HO-1 may represent potential therapeutic strategies for mitigating liver damage associated with paracetamol overdose^50^. Nrf2 serves as a transcription factor that modulates the expression of genes involved in antioxidant defense and cellular protection in response to oxidative stress. In the context of paracetamol-induced hepatotoxicity, activation of Nrf2 is crucial for safeguarding the liver against injury^51^. Upon activation, Nrf2 translocates into the nucleus in which it binds to AREs within the promoter regions of target genes. This includes genes such as HO-1, NAD(P)H quinone oxidoreductase 1 (NQO1), and GSTs. The binding of Nrf2 to AREs leads to the upregulation of these antioxidant enzymes and phase-II detoxification enzymes. Consequently, these enzymes play a crucial role in neutralizing ROS and detoxifying harmful metabolites generated during paracetamol metabolism^52^. HO-1 is an enzyme that catalyzes the degradation of heme into biliverdin, carbon monoxide (CO), and iron. Biliverdin is then converted into bilirubin, which possesses antioxidant properties. Additionally, CO exerts cytoprotective effects. HO-1 is induced by various stress stimuli, including oxidative stress, and its upregulation serves as a crucial adaptive response to paracetamol-induced hepatobiliary injury^49^. The degradation of heme by HO-1, resulting in the production of biliverdin and CO, contributes to antioxidant, anti-inflammatory, and anti-apoptotic effects. These mechanisms collectively help mitigate liver damage induced by paracetamol overdose.

## Conclusion

*Paederia foetida* treatment significantly suppressed the level of hepatic and non-hepatic parameters with alteration of glucose, lipid, and renal parameters. It suppressed the level of inflammatory cytokines and parameters and altered the mRNA expression of TNF-α, IL-1β, IL-6, IL-10, Bax, caspase-3, Bcl-2, Nrf_2_, HO-1, NQO-1, and NF-κB. *Paederia foetida* exhibited the hepatoprotective effect via inflammatory and Nrf2/HO-1 pathway.

## Data Availability

All the data will be available on the request to the corresponding author.

## References

[1] Mukhtar S, Xiaoxiong Z, Qamer S, Saad M, Mubarik MS, Mahmoud AH, Mohammed OB (2021). Hepatoprotective activity of silymarin encapsulation against hepatic damage in albino rats. Saudi J Biol Sci.

[2] Anantha KC, Siva RC, Manohar RA (2012). Hepatoprotective effect of biherbal ethanolic extract against paracetamol-induced hepatic damage in albino rats. J Ayurveda Integr Med.

[3] Gupta AK, Misra N (2006). Hepatoprotective activity of aqueous ethanolic extract of chamomile capitula in paracetamol intoxicated albino rats. Am J Pharmacol Toxicol.

[4] Igbudu UC, Christian AG, Chukwuemeka OP, Toochukwu OF, Onyebuchi AD, Tochukwu OA (2024). Hepatoprotective effect of the ethanol extract of Detarium senegalense stem bark in albino Wistar. Int J Biol Pharm Sci Arch.

[5] Vidhya Malar HL, Mary S, Bai M (2009). Hepato-protective activity of phyllanthus emblica against paracetamol induced hepatic damage in wister albino rats. African J Basic Appli Sci.

[6] Yanpallewar SU, Sen S, Tapas S, Kumar M, Raju SS, Acharya SB (2003). Effect of Azadirachta indica on paracetamol-induced hepatic damage in albino rats. Phytomedicine.

[7] Hinson JA, Roberts DW, James LP, Uetrecht J (2010). Handbook of Experimental Pharmacology.

[8] Athersuch TJ, Antoine DJ, Boobis AR, Coen M, Daly AK, Possamai L (2018). Paracetamol metabolism, hepatotoxicity, biomarkers and therapeutic interventions: A perspective. Toxicol Res.

[9] Zhang LQ, Nsumu M, Huang P, Heruth DP, Riordan SM, Shortt K, Zhang N, Grigoryev DN, Li DY, Friesen CA, Van Haandel L, Leeder JS, Olson J, Ye SQ (2018). Novel protective role of nicotinamide phosphoribosyltransferase in acetaminophen-induced acute liver injury in mice. Am J Pathol.

[10] Gao TH, Liao W, Lin LT, Zhu ZP, Lu MG, Fu CM, Xie T (2022). Curcumae rhizoma and its major constituents against hepatobiliary disease: Pharmacotherapeutic properties and potential clinical applications. Phytomedicine.

[11] Ichikawa M, Okada H, Nakamoto N, Taniki N, Chu PS, Kanai T (2024). The gut-liver axis in hepatobiliary diseases.

[12] Schroeder CA, Johnson RA, Snyder LBC, Schroeder CA (2021). Canine and feline anesthesia and co-existing disease.

[13] Kavanagh C, Shaw S, Webster CRL (2011). Coagulation in hepatobiliary disease. J Vet Emerg Crit Care.

[14] Song X, Cui W, Meng F, Xia Q, Li X, Hou M, Jia L, Zhang J (2022). Glucopyranose from Pleurotus geesteranus prevent alcoholic liver diseases by regulating Nrf2/HO-1-TLR4/NF-κB signalling pathways and gut microbiota. Food Function.

[15] Dai JM, Guo WN, Tan YZ, Niu KW, Zhang JJ, Liu CL, Yang XM, Tao KS, Chen ZN, Dai JY (2021). Wogonin alleviates liver injury in sepsis through Nrf2-mediated NF-κB signalling suppression. J Cell Mol Med.

[16] Shen B, Feng H, Cheng J, Li Z, Jin M, Zhao L, Wang Q, Qin H, Liu G (2020). Geniposide alleviates non-alcohol fatty liver disease via regulating Nrf2/AMPK/mTOR signalling pathways. J Cell Mol Med.

[17] Dutta PP, Marbaniang K, Sen S, Dey BK, Talukdar NC (2023). A review on phytochemistry of *Paederia foetida* Linn. Phytomedicine Plus.

[18] Kumar V, Anwar F, Ahmed D, Verma A, Ahmed A, Damanhouri ZA (2014). *Paederia foetida* Linn. leaf extract: An antihyperlipidemic, antihyperglycaemic and antioxidant activity. BMC Complement Altern Med.

[19] Osman H, Rahim AA, Isa NM, Bakhir NM (2009). Antioxidant activity and phenolic content of *Paederia foetida* and Syzygium aqueum. Molecules.

[20] Khamphaya T, Pouyfung P, Yimthiang S, Kotepui M, Kuraeiad S (2022). Ameliorative effects of *Paederia foetida* Linn. on lead acetate-exposed rats. J Appl Pharm Sci.

[21] Kumar V, Al-Abbasi FA, Ahmed D, Verma A, Mujeeb M, Anwar F (2015). *Paederia foetida* Linn. inhibits adjuvant induced arthritis by suppression of PGE2 and COX-2 expression via nuclear factor-κB. Food Function.

[22] Kumar V, Kaithwas G, Anwar F, Rahman M, Patel DK, Singh Y (2016). Effect of variable doses of *Paederia foetida* L. combat against experimentally- induced systemic and topical inflammation in wistar rats. Curr Bioact Comp.

[23] Barros PP, da Silva GH, Gonçalves GMS, Oliveira JC, Pagnan LG, Arco-e-Flexa L (2017). Hepatoprotective effect of quercetin pretreatment against paracetamol-induced liver damage and partial hepatectomy in rats. Braz Arch Biol Technol.

[24] Mohamad NE, Yeap SK, Lim KL, Yusof HM, Beh BK, Tan SW (2015). Antioxidant effects of pineapple vinegar in reversing of paracetamol-induced liver damage in mice. Chin Med.

[25] Hurkadale PJ, Shelar PA, Palled SG, Mandavkar YD, Khedkar AS (2012). Hepatoprotective activity of Amorphophallus paeoniifolius tubers against paracetamol-induced liver damage in rats. Asian Pac J Trop Biomed.

[26] Gu X, Manautou JE (2012). Molecular mechanisms underlying chemical liver injury. Expert Rev Mol Med.

[27] Chen S, Melchior WB, Wu Y, Guo L (2014). Autophagy in drug-induced liver toxicity. J Food Drug Analysis.

[28] Vargas-Mendoza N, Madrigal-Santillán E, Morales-González A, Esquivel-Soto J, Esquivel-Chirino C, García-Luna Y, González-Rubio M, Gayosso-de-Lucio JA, Morales-González JA (2014). Hepatoprotective effect of silymarin. World J Hepatol.

[29] Gillessen A, Schmidt HHJ (2020). Silymarin as supportive treatment in liver diseases: a narrative review. Adv Therapy.

[30] Jiang WP, Deng JS, Huang SS, Wu SH, Chen CC, Liao JC (2021). Sanghuangporus sanghuang mycelium prevents paracetamol-induced hepatotoxicity through regulating the mapk/nf-κb, keap1/nrf2/ho-1, tlr4/pi3k/akt and camkkβ/lkb1/ampk pathways and suppressing oxidative stress and inflammation. Antioxidants.

[31] Galal RM, Zaki HF, El-Nasr MMS, Agha AM (2012). Potential protective effect of honey against paracetamol-induced hepatotoxicity. Arch Iran Med.

[32] Gokkaya EO, Yesilot S, Ozgocmen M, Aslankoc R, Aydin Acar C (2022). Protective effects of resveratrol and avocado oil against paracetamol-induced hepatotoxicity in rats. Drug Chem Toxicol.

[33] Bouhlali EDT, Derouich M, Hmidani A, Bourkhis B, Khouya T, Filali-Zegzouti Y, Alem C (2021). Protective effect of Phoenix dactylifera L. seeds against paracetamol-induced hepatotoxicity in rats: a comparison with vitamin C. ScientificWorldJournal.

[34] Koshak MF, El-Readi MZ, Elzubier ME, Refaat B, Almaimani RA, Idris S (2023). Antioxidative and anti-inflammatory protective effects of fucoxanthin against paracetamol-induced hepatotoxicity in rats. Marine Drugs.

[35] Sayed AM, Hassanein EHM, Salem SH, Hussein OE, Mahmoud AM (2020). Flavonoids-mediated SIRT1 signaling activation in hepatic disorders. Life Sciences.

[36] Panche AN, Diwan AD, Chandra SR (2016). Flavonoids: An overview. J Nutrit Sci.

[37] Karak P. (2019). Biological activities of flavonoids: an overview. International Journal of Pharmaceutical Sciences and Research.

[38] Ugan RA, Cadirci E, Un H, Cinar I, Gurbuz MA (2023). Fisetin attenuates paracetamol-induced hepatotoxicity by regulating CYP2E1 enzyme. Anais Acad Bras Ciênc.

[39] Ruyani A, Sinta BD, Emilia Zulfikar, Anansyah F, Putri SR, Sundaryono A (2018). Preliminary studies on therapeutic effect of ethanolic extract of Tylophora villosa leaves against paracetamol-induced hepatotoxicity in mice. J Tradit Complement Med.

[40] Coelho AM, Queiroz IF, Lima WG, Talvani A, Perucci LO, Oliveira de Souza M (2023). Temporal analysis of paracetamol-induced hepatotoxicity. Drug Chem Toxicol.

[41] Levitt MD, Hapak SM, Levitt DG (2022). Alkaline phosphatase pathophysiology with emphasis on the seldom-discussed role of defective elimination in unexplained elevations of serum ALP - a case report and literature review. Clin Exp Gastroenterol.

[42] Devkar ST, Kandhare AD, Zanwar AA, Jagtap SD, Katyare SS, Bodhankar SL (2016). Hepatoprotective effect of withanolide-rich fraction in acetaminophen-intoxicated rat: decisive role of TNF-α, IL-1β, COX-II and iNOS. Pharm Biol.

[43] Jaeschke H, Knight TR, Bajt ML (2003). The role of oxidant stress and reactive nitrogen species in acetaminophen hepatotoxicity. Toxicol Lett.

[44] Mancuso C, Pani G, Calabrese V (2006). Bilirubin: An endogenous scavenger of nitric oxide and reactive nitrogen species. Redox Rep.

[45] Katawala T. (2008). Liver physiology.

[46] Nithiyanandam S, Prince SE (2023). Caesalpinia bonducella counteracts paracetamol-instigated hepatic toxicity via modulating TNF-α and IL-6/10 expression and Bcl-2 and caspase-8/3 signalling. Appl Biochem Biotechnol.

[47] Tan Q, Hu J, Yu X, Guan W, Lu H, Yu Y (2016). The role of IL-1 family members and Kupffer cells in liver regeneration. BioMed Res Int.

[48] Rao R, Graffeo CS, Gulati R, Jamal M, Narayan S, Zambirinis CP (2014). Interleukin 17-producing γδT cells promote hepatic regeneration in mice. Gastroenterology.

[49] Loboda A, Damulewicz M, Pyza E, Jozkowicz A, Dulak J (2016). Role of Nrf2/HO-1 system in development, oxidative stress response and diseases: an evolutionarily conserved mechanism. Cell Mol Life Sci.

[50] Gum S Il, Cho MK (2013). Recent updates on acetaminophen hepatotoxicity: The role of Nrf2 in hepatoprotection. Toxicol Res.

[51] Aleksunes LM, Manautou JE (2007). Emerging role of Nrf2 in Protecting against hepatic and gastrointestinal disease. Toxicol Pathol.

[52] Ngo V, Duennwald ML (2022). Nrf2 and oxidative stress: a general overview of mechanisms and implications in human disease. Antioxidants.

